# Genome assembly and annotation of microalga *Nannochloropsis oceanica* C018

**DOI:** 10.1128/mra.00883-24

**Published:** 2024-12-27

**Authors:** Adrian Estrada-Graf, Hari Koneru, Jason Arnold, Sara Calhoun, Igor V. Grigoriev, Zackary I. Johnson

**Affiliations:** 1 Marine Laboratory, Duke University, Beaufort, North Carolina, USA; 2 Department of Molecular Genetics and Microbiology, Duke Microbiome Center, Duke University, Durham, North Carolina, USA; 3 US Department of Energy Joint Genome Institute, Lawrence Berkeley National Laboratory, Berkeley, California, USA; 4 Department of Plant and Microbial Biology, University of California Berkeley, Berkeley, California, USA; 5 Biology, Civil & Environmental Engineering and Duke Microbiome Center, Duke University, Durham, North Carolina, USA; University of California Riverside, Riverside, California, USA

**Keywords:** algae, carbon capture, biofuels, next-generation sequencing, biotechnology, bioinformatics

## Abstract

The microalga *Nannochloropsis* is an important organism for algae-based biocommodity production of food, feed, and fuel, among other products. Using PacBio Revio, we sequenced, assembled, and annotated a 26.41 Mbp *Nannochloropsis oceanica* C018 genome.

## ANNOUNCEMENT

Microalgae-based technologies have emerged as sustainable approaches to help mitigate the impact of rising CO_2_ emissions while providing scalable solutions applicable in agriculture, medicine, and energy, among others. *Nannochloropsis* microalgae exhibit high productivity and tolerance for elevated CO_2_ concentrations, thrive in outdoor conditions, have potential for biofuel production, and have emerged as model organisms for applied phycology (1, 2). Additionally, the biochemical characteristics of *Nannochloropsis* make them a valuable candidate as a food source for both animals and humans. *Nannochloropsis oceanica* C018 was isolated by S Brown (University of Hawaii-Manoa) near the Island of Hawaii (19.666° -156.032°) using dilution to extinction with seawater-based F/2 medium (3).

*Nannochloropsis oceanica C018* was grown in 20 L carboys with F/2 (3) on a 12-hour day/night light cycle (~400 µmol Q/m^2^/sec) at 25°C and bubbled with sterile air. Following cell harvesting, DNA was isolated with Quick-DNA/RNA Miniprep Kit D7001 (Zymo Research, USA). Total DNA was quantified using DeNovix DS-11 Series Spectrophotometer/Fluorometer (DeNovix Inc., USA) and normalized to 45 ng/µL prior to sequencing library preparation. DNA fragments between 15 and 20 kb were selected for sequencing, and libraries were prepared using Express Template Prep kit version 3 (Pacific Biosciences, USA) for standard HiFi library preparation. High-fidelity sequencing reads were obtained with Pacific Biosciences Revio system with version SMRT Link v13 and Instrument Control Software (ICS) v13. 1,906,268 total reads were generated with an N50 value of 5090 bp, and HiFi read yield was 11,001,781,445 bp.

Genome assembly followed a modified version of the VGP pipeline (4) in usegalaxy.org (5). Samtools v.1.15.1 (6) was used to extract fastq files from .bam sequence output files, and Cutadapt v.4.6 (7) was implemented to trim adapter sequences and filter low-quality reads. Hifiasm v.0.19.8 (8) was used to assemble contigs from high-fidelity reads with “light” purge level. The resulting assembly was decontaminated with kraken2 v.2.1.1 (9), and duplicates were purged with purge_dups v.1.2.6 (10). Completeness of the resulting genome was estimated with BUSCO v.5.5.0 (11) and quality assessed with QUAST v.5.2.0 (12). Default parameters were used except where otherwise noted.

The resulting genome assembly consisted of 30 contigs, 26.41 Mbp (415× coverage) with a GC content of 54.26%, and a L_50_ of 10. The genome was annotated following the JGI Annotation pipeline and PhycoCosm (12), with 80 million paired-end transcriptomic reads. Functional annotation of the 8,768 gene models was performed using the JGI annotation pipeline. Both genome assembly size and gene count are within the range of other published *Nannochloropsis* genomes (13–15) (https://phycocosm.jgi.doe.gov/eustigmatophyta). This genome will spur the continued development of microalgae for use as a biofuel feedstock and provide prerequisite information needed for genetic manipulation and expression analyses.

## Data Availability

This whole genome project has been deposited at GenBank under the accession JBEBFO000000000. The version described in this paper is JBEBFO010000000. Raw sequencing data has been deposited in the NCBI Sequence Read Archive (SRA) under the BioProject accession PRJNA1118351. Genome assembly and annotations are also accessible at the JGI algal portal PhycoCosm (12): phycocosm.jgi.doe.gov/NanspC018_1/.
